# Emergency department 72-hour revisits among children with chronic diseases: a Saudi Arabian study

**DOI:** 10.1186/s12887-018-1186-8

**Published:** 2018-06-26

**Authors:** Anwar E. Ahmed, Bashayr I. ALMuqbil, Manair N. Alrajhi, Hend R. Almazroa, Doaa A. AlBuraikan, Monirah A. Albaijan, Maliha Nasim, Majid A. Alsalamah, Donna K. McClish, Hamdan AL-Jahdali

**Affiliations:** 1 King Abdullah International Medical Research Center (KAIMRC), Riyadh, Saudi Arabia; 2 King Saud bin Abdulaziz University for Health Sciences, National Guard Health Affairs, P.O. Box 22490, Riyadh, 11426 Saudi Arabia; 3 Department of Biostatistics, School of Medicine, Virginia Commonwealth University, Box 980032, Richmond, VA 23298 USA; 4 McGill University, Montreal, Canada; 5 Pulmonary Division Medical Director of sleep disorders, Center King Abdulaziz Medical City, Riyadh, Saudi Arabia

**Keywords:** Emergency department, ED revisit, Chronic diseases, Children, Saudi Arabia

## Abstract

**Background:**

Emergency Department (ED) revisits have often been used as an indicator of medical care quality. This study aimed to quantify the frequency of ED revisits within 72 h of discharge and identify its factors among children with chronic diseases.

**Methods:**

We designed a retrospective cohort study of children with at least one chronic disease who were also under 18 years of age and had attended and were discharged from the ED at King Abdullah Specialist Children’s Hospital (KASCH-RD), Riyadh, Saudi Arabia between April 19, 2015 and July 29, 2017. The outcome measure was the frequency of ED revisits during a period of 72 h after discharge.

**Results:**

The study included 11,057 ED discharges of children with at least one chronic disease. Their revisit rate was 1211 (11%), with 83 (6.9%) having had a second ED revisit within 72 h of ED discharge. According to ICD-10 codes, the most common causes of ED revisits were respiratory, digestive, genitourinary, symptoms, and external causes. Factors of frequent ED revisits within 72 h were young age, institutional health insurance coverage, year of new health information system (2015), external causes, and genitourinary.

**Conclusion:**

The rate of 72-h ED revisits after discharge of children with chronic diseases treated at KASCH-RD was relatively high, and was associated with young age, institutional health insurance coverage, year of a new health information system implementation, and external causes of ED visit. These study findings amplify the need for intervention to reduce the rate of early ED revisits among children with chronic diseases.

## Background

Overcrowding in pediatric emergency departments (EDs) places a heavy burden on healthcare systems in terms of financial costs [1, 2] and potential infection-related ED visits [3]. Recently, significant interest and research has focused on the number of return-to-emergency department (ED) visits, as it represents a quality benchmark for patient safety and care [4–6]. This number also contributes to overcrowding in EDs, as some visits are unnecessary [4, 7].

ED visits were observed in patients of various age groups, with a large amount having been noticed in children [8]. Earlier studies have assessed the rate of ED revisits among children in general: it ranges between 2.7 and 19% [9–17]. However, a high frequency of ED revisits was observed in children with chronic diseases [14, 15]. The large variation rates of ED revisits among children highlights the need for more evaluation, particularly in unstudied populations.

In several international reports, the greater rate of ED revisits in children has been attributed to young age [9, 12, 15, 18] and health insurance coverage [18–20]. Recent Saudi Arabian studies, however, have documented that chronic diseases are the top cause of death among children in Saudi Arabia [21, 22], and have become a major priority in Saudi Vision 2030.

Despite the fact that revisits to the ED are well recognized as a key for quality improvement [4–6], no data exists in Saudi Arabia regarding the rate of ED revisits among children with chronic diseases. This study attempts to investigate the frequency of ED revisits at a major Saudi hospital and the common causes, and to identify factors among children with chronic diseases associated with the high rate of 72-h ED revisits after discharge. We assessed the hypothesis that younger children, health insurance coverage, year of a new health information system implementation, and causes of ED visits may be associated with the high rate of ED revisits within 72 h among the study population.

## Methods

This is a retrospective study of all ED discharges of children with chronic diseases under the age of 18 years who attended the ED at King Abdullah Specialist Children’s Hospital, Riyadh (KASCH-RD) between April 19, 2015 and July 29, 2017. The study obtained approval from the Institutional Review Board (IRB) at the Ministry of National Guard - Health Affairs (MNG-HA), approval #RC17/081/R.

The ED records of eligible children were retrieved from the institution’s BESTCare database. In 2015, the children’s hospital implemented a new integrated health information system, BESTCare. It is a unified database that allows integration of all main hospital units: inpatient, outpatient, emergency, intensive care, and operating room. All medical documentation, medical orders, medication histories, and radiology and lab results are stored in chronological order [23]. Housing a wide range of administration and clinical activity (including a clinical data warehouse), it also benefits from a clinical decision support system that highlights standardized clinical guidelines of procedures to be followed for certain conditions, and issues alerts to physicians, on ordering investigations or prescribing medication that may be contraindicated or unsafe [23]. We defined the study population as children with at least one chronic disease who were discharged from the ED at KASCH-RD during the study period. We included the main types of chronic diseases that require long-term control [24]. These chronic diseases were defined by the Saudi Ministry of Health as national health priorities for prevention and treatment, such as cardiovascular disease, diabetes mellitus, hypertension, cancer, pulmonary disease, and asthma [25].

The study data included age, gender, institutional health insurance status (yes/no), new patient or patient has not *received* healthcare services in our facility (yes/no), year of ED visit (2015, 2016, or 2017). Patients’ ages were classified into 4 groups: < 3 years, 3 ≤ age < 6 years, 6 ≤ age < 14 years, and ≤ 14 age < 18 years old.

In our center the health coverage includes 3 categories: 1) Ministry of National Guard employees and their dependents who are fully covered. 2) those with private health insurance coverage and 3) those without insurance but covered exceptionally based on their case complexity. We reclassified health insurance status into two groups; institutionally insured or privately insured. In our center the emergency department is open to all emergency cases. Case that sever or need urgent admission will be admitted to our hospital regardless of their insurance coverage, or nationalities.

The study units of analysis are ED discharges of children with at least one chronic disease. The study outcome was the number of ED revisits within 72 h of discharge (0,1,2 etc). The causes of initial ED visit/revisits were classified according to the International Statistical Classification of Diseases and Related Health Problems (10th version, Australian modification) code, chapters I “Certain infectious and parasitic diseases” to XXII “Codes for special purposes” [26]. The ICD-10 is publically available at http://apps.who.int/classifications/icd10/browse/2016/en#/XVIII.

### Data analysis

**Fig. 1:**
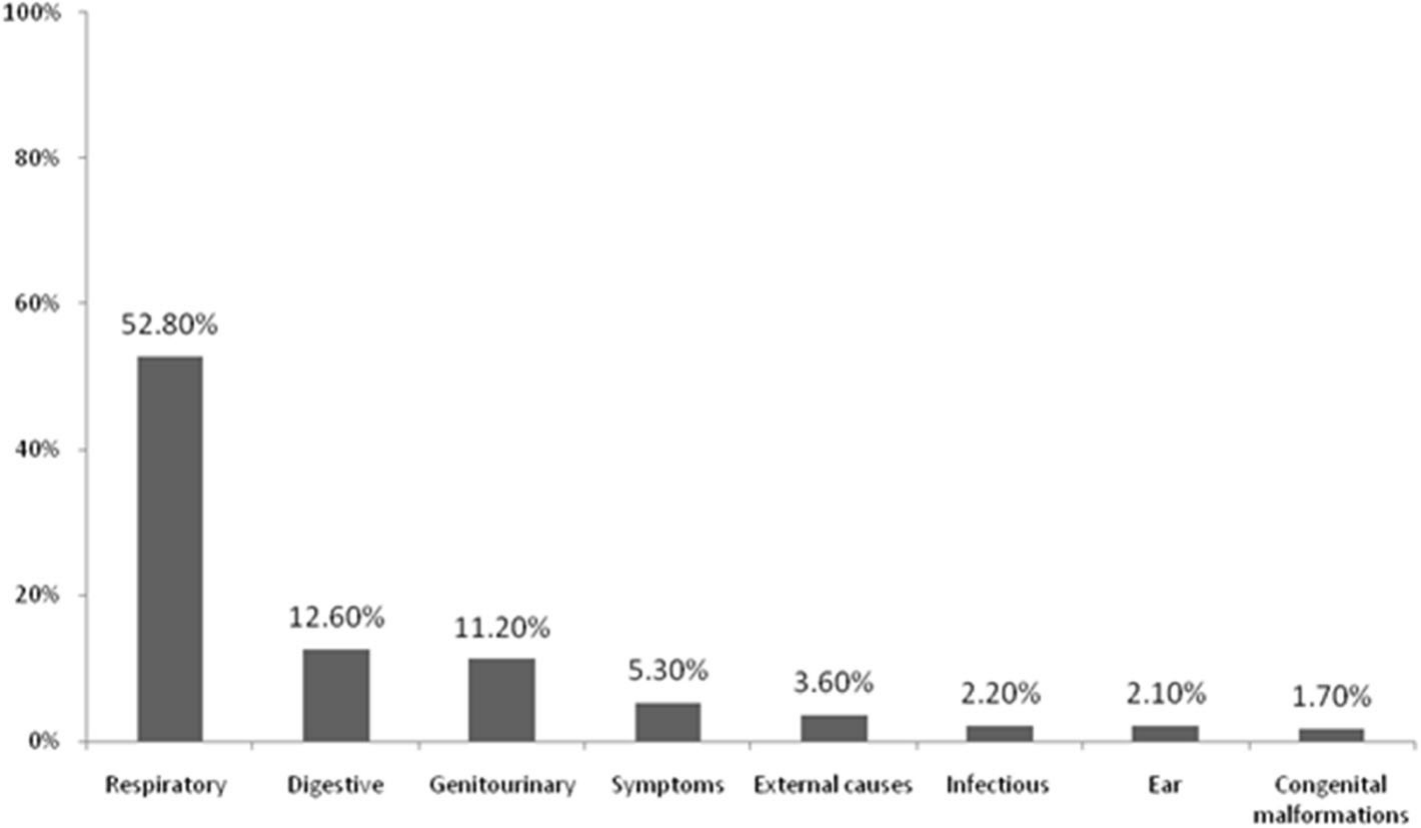
The most common causes at the first ED revisit within 72 h of discharge

**Fig. 2:**
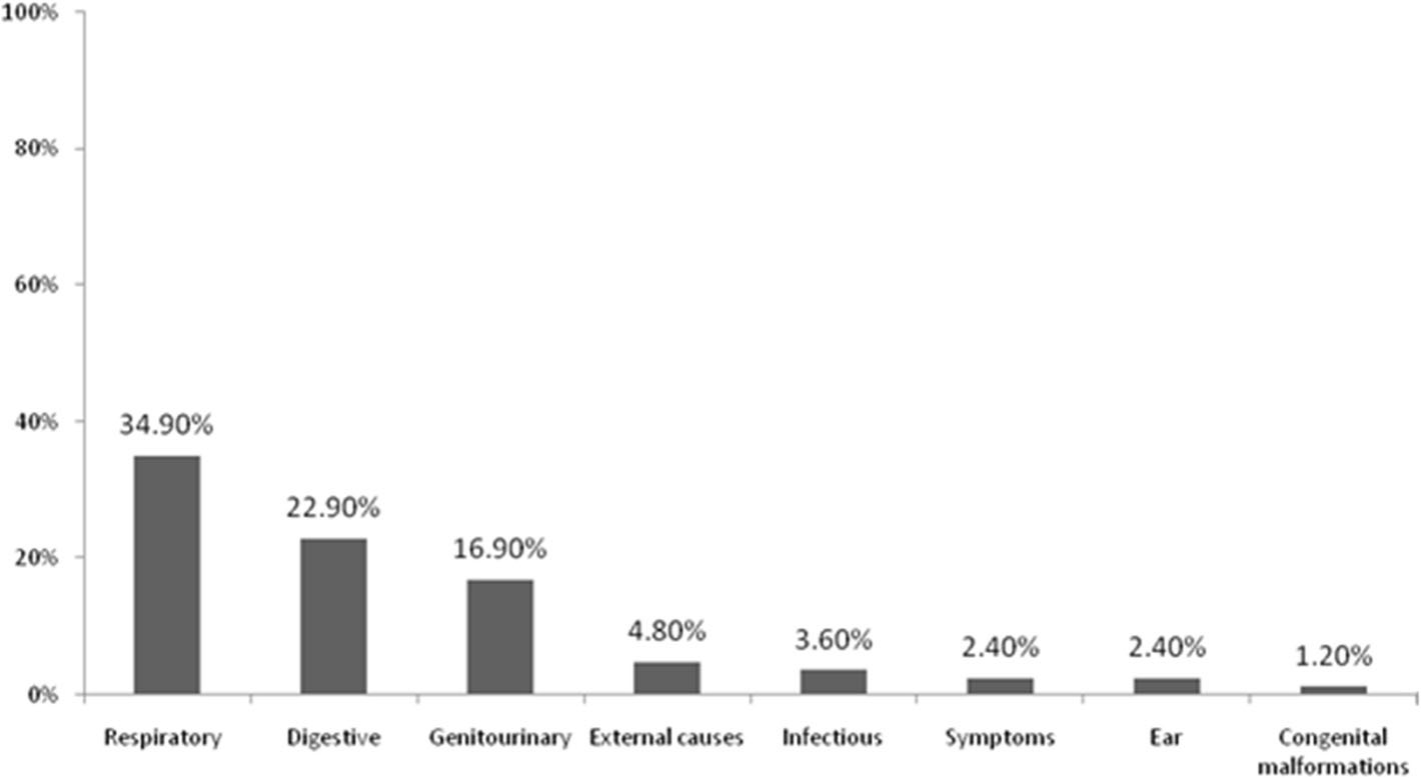
The most common causes at the second ED revisit within 72 h of discharge

The data analysis was performed using IBM SPSS v. 25 (IBM Corp., Armonk, NY, USA). Subject characteristics were illustrated as frequency and percentage (Table 1). The most common causes reported at the first ED revisit and the second ED revisit within 72 h were presented in bar charts (Figs. 1 & 2). The bar charts were generated using Microsoft Excel 2010. A univariate Poisson regression model was used to calculate the unadjusted relative rate (uRR) and assess differences in the rate of ED revisits within 72 h of ED discharge across children’s characteristics. Multiple Poisson regression models were used to calculate the adjusted relative rate (aRR) and identify independent factors that were associated with the high rate of ED revisits within 72 h of ED discharge. Table 2 shows the findings of Poisson regression models: *p*-value (P), RR, and confidence intervals (CI) for RR. A *P* ≤ 0.05 was considered significant.

**Table 1:** Characteristics of ED discharges at KASCH-RD between September 13, 2015 and July 29, 2017

Characteristics	Levels	Number	Percentage
Age	< 3 years	2735	32.1
	3 ≤ Age < 6 years	2192	25.8
	6 ≤ Age < 14 years	3151	37.0
	≥14 years	432	5.1
Gender	Female	3394	39.9
	Male	5116	60.1
Institutional health insurance coverage	Yes	8223	96.6
	No	287	3.4
Year of ED visit	2015	2538	23.0
	2016	5663	51.2
	2017	2856	25.8
New patient ED visit	Yes	533	17.4
	No	2526	82.6
Causes of initial ED visit	Circulatory	362	3.3
	Congenital malformations	371	3.4
	Digestive	2046	18.5
	Ear	478	4.3
	External causes	851	7.7
	Genitourinary	1954	17.7
	Respiratory	2606	23.6
	Other	2389	21.6
Initial ED discharges (*N* = 11,057)			
Had first revisit within 72 h	No	9846	89.0
	Yes	1211	11.0
Had a second revisit within 72 h	No	1128	93.1
	Yes	83	6.9

**Table 2:** Factors associated with higher rate of ED revisits within 72 h of discharge

		Univariate				Multivariate			
				95% Wald CI for RR				95% Wald CI for aRR	
Factor	Reference	P	RR	Lower	Upper	P	aRR	Lower	Upper
Age < 3 years	*≥14 years*	0.001*	3.293	2.103	5.158	0.001*	6.056	2.461	14.904
3 ≤ Age < 6 years	*≥14 years*	0.001*	3.114	1.982	4.893	0.001*	4.831	1.930	12.091
6 ≤ Age < 14 years	*≥14 years*	0.001*	2.392	1.524	3.754	0.036*	2.663	1.068	6.641
Female	*Male*	0.830	1.013	0.898	1.143	0.094	1.253	0.962	1.633
Institutional health insurance coverage	*None*	0.001*	4.239	2.200	8.168	0.025*	3.132	1.152	8.518
Year 2015	*Year 2017*	0.001*	1.363	1.166	1.593	0.001*	2.040	1.390	2.993
Year 2016	*Year 2017*	0.001*	1.288	1.124	1.475	0.050*	1.427	1.000	2.037
New patient ED visit	*No*	0.341	0.833	0.572	1.213	0.950	0.988	0.681	1.434
Circulatory	*Other*	0.687	1.075	0.756	1.529	0.595	0.858	0.487	1.511
Congenital malformations	*Other*	0.296	1.195	0.856	1.667	0.102	1.401	0.935	2.099
Digestive	*Other*	0.001*	1.490	1.249	1.777	0.141	1.456	0.883	2.399
Ear	*Other*	0.026*	1.380	1.039	1.832	0.518	1.217	0.671	2.204
External causes	*Other*	0.449	0.902	0.690	1.178	0.039*	1.944	1.034	3.657
Genitourinary	*Other*	0.001*	1.765	1.487	2.095	0.002*	1.814	1.251	2.631
Respiratory	*Other*	0.001*	1.435	1.212	1.699	0.001*	0.111	0.035	0.351

*. Significant at α = 0.05

## Results

The KASCH-RD ED medical records of 11,057 ED discharges for children with chronic diseases were analyzed. Of the ED discharges, 1211/11,057 (11%) had a first ED revisit, and 83/1211 (6.9%) had a second ED revisit within 72 h. The median age was 4.9 (25 percentile = 2.3 and 75 percentile = 8.7 years), with 60.1% being males. Among the ED discharges, 618 (5.6%) children were admitted to the hospital. Table 1 summarizes the sample characteristics. Among children studied, the 6 most common chronic diseases were asthma (8.9%), allergy (3.6%), heart disease (1.6%), eczema (1%), anemia (0.6%), and diabetes (0.2%).

The most common causes at the initial ED visit were respiratory (23.6%), digestive (18.5%), genitourinary (17.7%), and external causes of morbidity and mortality (7.7%). The most common causes on the first ED revisit were respiratory (52.8%), digestive (12.6%), genitourinary (11.2%), symptoms, signs, and abnormal clinical and laboratory findings (5.3%), and external causes of morbidity and mortality (3.6%) (Fig. 1). The most common causes on the second ED revisit were respiratory (34.9%), digestive (22.9%), genitourinary (16.9%), external causes of morbidity and mortality (4.8%), along with symptoms, signs, and abnormal clinical and laboratory findings (2.4%) (Fig. 2).

The results of unadjusted and adjusted Poisson analyses of the number of revisits within 72 h after ED discharge are illustrated in Table 2. The unadjusted relative risk (uRR) significantly increased in children with chronic diseases of age <  3 years (uRR = 3.293, *P* = 0.001), 3 ≤ age < 6 years (uRR = 3.114, *P* = 0.001), and 6 ≤ age < 14 years (uRR = 2.392, *P* = 0.001) compared to children with chronic diseases of age ≥ 14 years. Among children with chronic diseases, institutional health insurance coverage was associated with a higher frequency of revisits within 72 h (uRR = 4.239, P = 0.001). The risk of revisits within 72 h was higher for children with chronic diseases who visited the ED in 2015 (uRR = 1.363, *P* = 0.001) and 2016 (uRR = 1.288, *P* = 0.001) compared to children who visited the ED in 2017. Causes for ED visits, such as digestive diseases (uRR = 1.490, *P* = 0.001), genitourinary diseases (uRR = 1.765, *P* = 0.001), respiratory diseases (uRR = 1.435, *P* = 0.001), and ear diseases (uRR = 1.380, *P* = 0.026) were also significant predictors for a higher frequency of revisits within 72 h.

Independent risk factors for ED revisits within 72 h included age, year, institutional health insurance coverage, external causes, and genitourinary diseases (Table 2). Children with chronic diseases of a younger age were associated with a higher risk of ED revisits within 72 h: age <  3 years (aRR = 6.056, *P* = 0.001), 3 ≤ age < 6 years (aRR = 4.831, *P* = 0.001), and 6 ≤ age < 14 years (aRR = 2.663, *P* = 0.036) compared to children of age ≥ 14 years. The rate of ED revisits within 72 h was higher for children with institutional health insurance than for children without health insurance (aRR = 3.132, *P* = 0.025). The year of implementing a new health information system (2015) significantly predicted a high rate of ED revisits within 72 h (aRR = 2.040, *P* = 0.001), and the following year 2016 (aRR = 1.427, *P* = 0.050), compared to 2017. Among children with chronic diseases, external causes of morbidity and mortality (aRR = 1.944, *P* = 0.039) and genitourinary (aRR = 1.814, *P* = 0.002) were associated with a higher rate of revisits within 72 h after ED discharge. Gender was not associated with 72-h ED revisits among children with chronic diseases.

## Discussion

In this retrospective study, we included all ED discharges of patients with chronic diseases under the age of 18 years who were admitted to the ED at KASCH-RD between April 19, 2015 and July 29, 2017. This represents the first Saudi Arabian evaluation of children with chronic diseases re-attending the ED within 72 h after ED discharge. Our main aim was to determine the frequency of ED revisits and the main causes, as well as to identify characteristics associated with the high rate of 72-h ED revisits among children with chronic diseases.

A rather high ED revisit rate (11%) was observed among children with chronic diseases in Saudi Arabia as compared to the *general global children’s population*: USA 2.7% [9], Singapore 4.3% [10], Canada 4.4% [11], and Taiwan 6.47% [12]. It may not be possible to compare this study’s findings with these studies, as in our study we calculated the rate of ED revisit in a definitive group, specifically children with chronic diseases. However, this comparison indicates that the presence of chronic diseases is associated with a high rate of ED utilization.

A similar ED revisit rate was found in earlier investigations: two studies among young children with gastroenteritis [13] and common illnesses [15] showed 16%. In our study, we evaluated revisits within 72-h of ED discharge, and it should be noted that these two studies have evaluated longer time spans of ED revisits. For instance, Freedman et al. [13] evaluated revisits within 7 days.

Reducing early ED revisits at KASCH-RD must be a hospital priority to reduce unnecessary costs [16] and improve quality of services [17]. Understanding the reasons for ED revisits among children with chronic diseases, based on the initial ED visit discharge, may allow implementing intervention or guidelines to reduce the ED revisit rates. To reduce avoidable ED revisits, this could involve applying a predetermined framework on early follow-up and parent education on home management.

In the KASCH-RD center, respiratory conditions were found to be the most frequent cause of initial ED visits. This is in agreement with Goh et al., where it reported that respiratory conditions were linked with higher ED utilization [10]. Focusing on ED revisits related to respiratory diseases may reduce ED revisit rates by identifying non-urgent ED visits.

In this large cohort study, we noted that the rate of ED revisits decreased with age in children with chronic diseases. The greater ED revisit rate in younger children noted in the study is in agreement with earlier studies showing that children of a younger age are associated with frequent ED revisits [9, 12, 15, 18]. Interventions are needed to reduce ED revisits in younger children, such as referrals or early follow-up appointments in the outpatient clinic setting to specialty clinics, and parent involvement in the ED discharge process.

In this study, institutional health insurance coverage was found to be an important determinant of ED revisits in children with chronic diseases. In several reports, public health insurance coverage as compared with uninsured (self-pay) or privately insured has been cited as an important predictor of ED revisits. Walsh-Kelly et al. [18] noted that public insurance was associated with greater ED revisits. Scales et al. [19] and Jacobstein et al. [20] reported that the odds of ED revisits were 52 and 86% higher in the Medicaid group as compared to the private insurance group, respectively. The impact of institutional health insurance coverage on ED revisits in children with chronic diseases must be evaluated to identify preventable reasons for ED revisits.

A higher rate of ED revisits in children with chronic diseases appeared during the year 2015 as compared to the more recent year of 2017. This indicates that the rate of ED revisits in the Saudi facility decreased with time. This may be due to the 2015 implementation in the KASCH-RD facility of a new computerized hospital system, BESTCare, which is a unified electronic health information system that includes a clinical decision support system. The reduction of the ED revisit rate over time could be due to implementation of a wide array of training programs to promote and evaluate the use BESTCare among physicians.

Studies have shown that electronic health information systems, particularly those that incorporate clinical decision tools, have the advantage of improving the healthcare provider’s adherence to evidence-based clinical guidelines and care protocols [27, 28]. Improvements in adherence can lead to improved clinical outcomes [27–31] and potentially better quality of patient care, which may help to explain the reduction in ER revisits. However, it should be noted that these studies have taken place at inpatient and outpatient settings and not the ER, and thus have not directly assessed the effect of clinical decision tools.

The authors identified several limitations to the study. Although the number of ED visits studied was high, the findings were based on a single-center and retrospective study rather than a multi-center and prospective assessment of within 72 h of ED revisits. We did not record ED revisits occurring at another health facility. Furthermore, the study has not collected data on important details such as mode of arrival to the ED, number of chronic diseases, physician-related causes, and patient-related causes. Despite these limitations, to our knowledge, this study represents an initial investigation on early ED revisits and their causes among Saudi Arabian children with chronic diseases.

## Conclusions

A high rate of early ED revisits was found at KASCH-RD among children with chronic diseases, occurring frequently in one in ten children. In children with chronic diseases, higher ED revisit rates are associated with young age, institutional health insurance coverage, and causes for ED visits. Intervention could be implemented to examine whether parent education on home management, early follow-up appointment in an outpatient clinic setting, and clear discharge guidance could reduce the rate of early ED revisits among children with chronic diseases.

## Abbreviations

aRR: Adjusted relative rate
CI: Confidence interval
ED: Emergency Department
IRB: *Institutional Review Board*
KASCH-RD: King Abdullah Specialist Children’s Hospital, Riyadh
MNG-HA: Ministry of National Guard - Health Affairs
P: *p*-value
uRR: Unadjusted relative rate
